# Infected Baerveldt Glaucoma Drainage Device by *Aspergillus niger*

**DOI:** 10.1155/2015/249419

**Published:** 2015-05-07

**Authors:** Nurul-Laila Salim, Yaakub Azhany, Zaidah Abdul Rahman, Roziawati Yusof, Ahmad Tajudin Liza-Sharmini

**Affiliations:** ^1^Department of Ophthalmology, School of Medical Sciences, Universiti Sains Malaysia, Health Campus, 16150 Kubang Kerian, Kelantan, Malaysia; ^2^Hospital Universiti Sains Malaysia, 16150 Kubang Kerian, Kelantan, Malaysia; ^3^Department of Medical Microbiology & Parasitology, School of Medical Sciences, Universiti Sains Malaysia, Health Campus, 16150 Kubang Kerian, Kelantan, Malaysia

## Abstract

Fungal endophthalmitis is rare but may complicate glaucoma drainage device surgery. Management is challenging as the symptoms and signs may be subtle at initial presentation and the visual prognosis is usually poor due to its resistant nature to treatment. At present there is lesser experience with intravitreal injection of voriconazole as compared to Amphotericin B. We present a case of successfully treated *Aspergillus* endophthalmitis following Baerveldt glaucoma drainage device implantation with intravitreal and topical voriconazole.

## 1. Introduction

With recent advances in the usage of glaucoma drainage devices (GDD) for filtering surgery, it is currently more widely used for cases of refractory glaucoma. GDD comprise a tube that shunts aqueous humor directly to a reservoir plate, thus assisting in aqueous outflow. They are further divided into valved and nonvalved implants, depending on whether or not a valve mechanism is present to limit aqueous outflow if the intraocular pressure becomes too low. Baerveldt implant is a type of nonvalved GDD made of barium impregnated, rounded silicone with surface area of 250–350 mm^2^ and fenestrations at its plate. GDD is generally indicated when conventional trabeculectomy is unlikely to be successful. One of the most dreaded complications from ocular surgery includes postoperative endophthalmitis, which can occur even years after the surgery.

Most cases of bacterial endophthalmitis patients will present with eye pain and redness, besides reduction of vision. However, patients with fungal endophthalmitis may present with painless reduced vision and subtle eye redness. Thus, a high index of suspicion should be present, particularly in cases with multiple or complicated surgery such as history of exposed GDD tube.

Endophthalmitis secondary to fungal infection is rare. However, it had been shown to be more resistant to treatment with consequent poor visual prognosis. At present, there is relatively less experience with the usage of intravitreal voriconazole. We report a case of successfully treated infected GDD caused by* Aspergillus niger* with intravitreal and topical voriconazole along with removal of GDD.

## 2. Case Report

A 42-year-old carpenter with no other known previous medical illness first presented with right eye undiagnosed primary angle closure glaucoma (PACG) which had complicated with central retinal artery occlusion (CRAO) and secondary neovascular glaucoma (NVG). The right eye vision was 6/60, and left eye 6/6. The intraocular pressure (IOP) at presentation was 67 mmHg over the right eye and 16 mmHg over the left. He was managed with panretinal laser photocoagulation and intravitreal ranibizumab injection. The left eye was noted to have narrow angle with cup to disc ratio of 0.6. He was thus treated as left eye primary angle closure suspect (PACS). Laser peripheral iridotomy was done to both eyes.

Subsequently, right eye augmented trabeculectomy was done twice but failed to control the IOP. His IOP ranged between 38 and 45 mmHg with maximum topical antiglaucoma drugs. He was thus subjected to Baerveldt glaucoma drainage device (GDD) implantation with scleral patch. Irradiated scleral patch was used during the implantation of Baerveldt GDD and subconjunctival dexamethasone and gentamicin was given at the end of surgery. Postoperatively, he was discharged well with topical prednisolone acetate 1% and topical ciprofloxacin. The IOP was maintained at around 8 mmHg.

Six months following GDD implantation, the GDD tube was exposed. The visual acuity was maintained at hand movement with no evidence of uveitis or vitritis. He was managed with amniotic patch and resuturing of conjunctiva. The surgery was combined with cataract extraction and synechiolysis as he had already developed secondary cataract. Swab and culture were taken and showed negative result. Postoperatively, his intraocular pressure was reduced to around 8 mmHg without topical antiglaucoma. He was discharged well with topical prednisolone acetate 1% and ciprofloxacin every 2 hours.

A month later, he reported a painless decrease of vision associated with mild eye redness. His vision dropped from hand movement to projection of light. Ocular examination showed reexposed GDD tube associated with yellowish discharge. Anterior segment examination showed hazy cornea with dense fibrin in the anterior chamber. The IOP was 0 mmHg. B-scan revealed dense vitreous opacity and choroidal detachment. He was thus diagnosed clinically with endophthalmitis, which necessitates urgent vitreous tapping along with broad-spectrum antibiotics injection.

**Figure 1:**
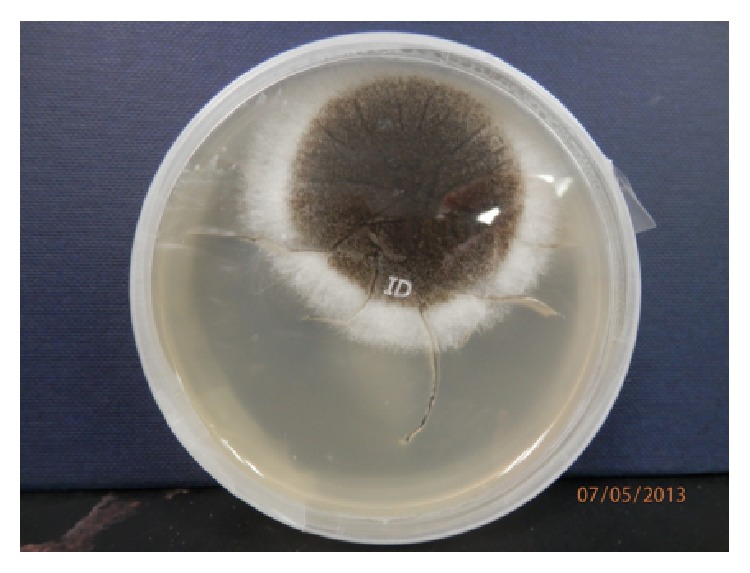
The surface colonies of the fungi on cultured plate consist of a compact white yellow basal felt covered by a dense layer of dark brown to black conidial heads and white on the reverse.

**Figure 2:**
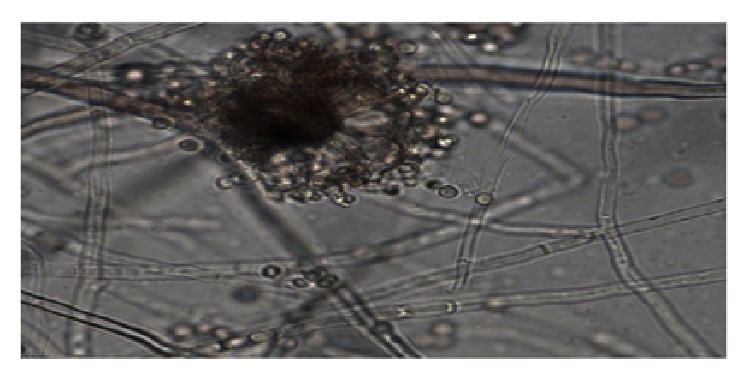
Wet mount preparation using lactophenol cotton blue was done and observed under light microscope. Presence of long and smooth conidiophores with black conidial heads was noted.

Diagnostic vitreous tap was done under aseptic technique and the specimen was sent for culture and antimicrobial susceptibility testing. He was treated with broad-spectrum antibiotics intravitreal injection of amikacin and vancomycin at the same setting. The* Aspergillus niger* was isolated on the third day based on colony morphology and characteristics features under light microscope (Figures 1 and 2).

Removal of the Baerveldt GDD was done subsequently as the eye was hypotony and the implant may serve as reservoir of infection, and he was treated with intravitreal voriconazole at the same setting. Topical voriconazole was also given in tapering dose over 5-month duration. At final assessment, his condition clinically improved with visual acuity of hand movement and the IOP remains below 6 mmHg. The anterior chamber reaction and vitritis also subsided.

## 3. Discussion

Glaucoma drainage devices are currently more widely used for cases of refractory glaucoma or cases of complicated glaucoma in which high risk of failure from conventional filtering surgery is anticipated.

One of the most dreaded complications from the surgery includes postoperative endophthalmitis, which can occur even years after the surgery. Exposed tube secondary to conjunctiva erosion appeared to be the main risk factor for development of infected glaucoma drainage device [1].

Other risk factors include history of leaking bleb, usage of antimetabolites, and placement of bleb inferior to horizontal median. Subsequent manipulations such as repositioning of the GDD tube, capsulectomy, and needling also carry with them higher risk for postoperative endophthalmitis [1, 2].

The commonest organism that may complicate trabeculectomy includes streptococci in acute endophthalmitis and both streptococci and* Haemophilus influenzae* in delayed endophthalmitis. Other organisms that may cause posttrabeculectomy endophthalmitis include other gram-negative organisms [1].

Fungal infection had rarely been implicated as the causative agent that may complicate postglaucoma surgery endophthalmitis. Fungal endophthalmitis in itself is infrequent.

The diagnosis of* Aspergillus* endophthalmitis is comparatively more difficult as there is no reliable serologic test available and blood culture is almost always negative. However, vitreous culture appears to yield high percentage of positive result and may help in establishing diagnosis.

The prognosis of fungal endophthalmitis depends upon the virulence of the organism, the timing of intervention, and the extent of intraocular involvement but generally results in poor visual outcomes [3].

This patient was suspected of fungal endophthalmitis in view of the slow and subtle presentation and eventually severe vitritis. He did not respond to powerful topical antibiotics. He was also on steroid at the same time as part of his postoperative treatment to reduce inflammation.

In this case, we removed the GDD as it was twice exposed despite surgical intervention of amniotic patching and it was highly suspicious of being the reservoir of infection besides not serving its purpose as the eye had become hypotony and signs of neovascular glaucoma had already subsided.

In literatures, there are differing opinions regarding removal of GDD following endophthalmitis. While some studies had shown that there appeared to be no significant difference in final visual acuity whether the implant was removed at the time of treatment or not, some other studies recommended the removal of GDD as it serves as reservoir of infection [2].

Treating fungal endophthalmitis is challenging as the visual prognosis is generally poor. At present, there is lesser experience with intravitreal injection of voriconazole as compared to Amphotericin B [4]. The first usage of intravitreal voriconazole in human eye for the treatment of endogenous endophthalmitis had been described by Kramer et al. in 2006 [5].

We report a rare case of fungal endophthalmitis following glaucoma drainage device implantation in an immunocompetent patient caused by* Aspergillus niger* which had been successfully treated with intravitreal and topical voriconazole.

## 4. Conclusion

Fungal endophthalmitis is rare but may complicate trabeculectomy with glaucoma drainage device, particularly in exposed tube, and it should be suspected in cases with painless decrease of vision. In this case, intravitreal and topical voriconazole along with removal of GDD had been shown to be effective in treating* Aspergillus niger* endophthalmitis.

## References

[1] Farber N., Muir K. (2012). Endophthalmitis after glaucoma surgery. *Eyenet Magazine*.

[2] Al-Torbak A. A., Al-Shahwan S., Al-Jadaan I., Al-Hommadi A., Edward D. P. (2005). Endophthalmitis associated with the Ahmed glaucoma valve implant. *British Journal of Ophthalmology*.

[3] Chhablani J. (2011). Fungal endophthalmitis. *Expert Review of Anti-Infective Therapy*.

[4] Riddell J., Comer G. M., Kauffman C. A. (2011). Treatment of endogenous fungal endophthalmitis: focus on new antifungal agents. *Clinical Infectious Diseases: Reviews of Anti-Infective Agents*.

[5] Kramer M., Kramer M. R., Blau H., Bishara J., Axer-Siegel R., Weinberger D. (2006). Intravitreal voriconazole for the treatment of endogenous *Aspergillus* endophthalmitis. *Ophthalmology*.

